# Supplementary material for: Characterization of the ddt1 Mutant in Rice and Its Impact on Plant Height Reduction and Water Use Efficiency
Source: Int J Mol Sci. 2024 Jul 11;25(14):7629. doi: 10.3390/ijms25147629 (PMC11277124; doi:10.3390/ijms25147629)
Supplement: Supplementary file 1 [file ijms-25-07629-s001.zip › Figure S1.pdf]

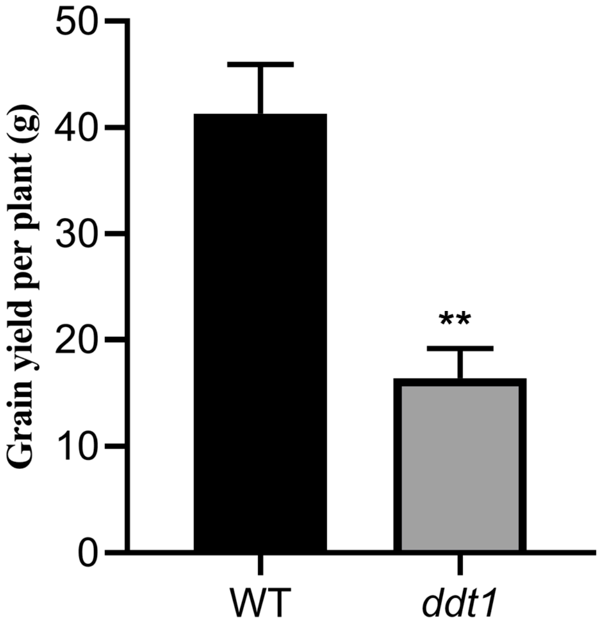

Figure S1. Grain yield per plant in WT and *ddt1*. ** Indicates statistical significance determined by student t-test, n = 10.
