# Supplementary material for: Characterization of the ddt1 Mutant in Rice and Its Impact on Plant Height Reduction and Water Use Efficiency
Source: Int J Mol Sci. 2024 Jul 11;25(14):7629. doi: 10.3390/ijms25147629 (PMC11277124; doi:10.3390/ijms25147629)
Supplement: Supplementary file 1 [file ijms-25-07629-s001.zip › Tabel S1. Prime used in the study.pdf]

Table S1. Prime used in the study.

Prime Name	Primer Sequence F (5'-3')	Primer Sequence R (5'-3')	Description
DDT1-Com	acgaattcgagctcggtaccAGAAACTAGTGCACAACGAAACA	tcgactctagaggatccGGGGAAAAGGAGGTAGTGCC	infusion into pCAMBIA-1300
Actin	catcttggcatctctcagcac	aactttgtccacgctaatgaa	qRT-PCR
DDT1-RT	GAAACACAGAGCCGATGACGA	GCAGGAGCTTCTGCTCGA	qRT-PCR
OsGA20ox3-RT	GTGGAAGGAGACCATGTCGTTCAACTGC	TCATCACGTCGCAGTACTCCTGGTACAC	qRT-PCR
OsGA20ox2-RT	CCCTCACCATCCTCCTCCAG	GGCAGCTCTTATACCTCCCGT	qRT-PCR
SLR1-RT	GATCGTCACCGTGGTAGAGC	GAGGGAATCGAACATGGTGG	qRT-PCR
APX1-RT	AGAGTCAGTACGATCAAGAC	TCTTGACAGCAAATAGCTTGG	qRT-PCR
POD1-RT	ACGTCGGGGTCGCCAACAAC	CGAACTCGTCCACCGACGCC	qRT-PCR
FeSOD-RT	CGACGCCGAGGAATTTCTAG	AGGTGGTGTAAGTGTCTCTCATGC	qRT-PCR
CATB-RT	GCTTGCTTTCTGCCCAGCGATAAT	AAATAGTTTGGGCCAAGACGGTGC	qRT-PCR
B3-1	GGGCTGTCATTGTCACGAG	GGCATCGACTCATCAGCC	Mapping
B3-3	TCGTCCATCCATTGATGCTAATC	TGCCATTTATCATTTGCCATTC	Mapping
P1	TCGAGTATCGATCATCTGCT	ATCGAGATGTATGCAAGGAT	Mapping
P2	CATGTCCTCACTTTAACCCC	TCTTGAATCCCTCTCACAGA	Mapping
P3	GGAGTACTCGATTCAAGTGG	GCTGTTGTTTGAGAGTTGAG	Mapping
P4	ATGGCTTCGTTTCACCG	TTACTCTGCACAGATGGATG	Mapping
P5	TAGCCTGTAGGGACTGTAAG	GTAGAAGTTCTCACTACCGC	Mapping
P6	TCTCAGACAGCAAAAGACAAT	TGTTACCATAGCATCGTCG	Mapping
